# Ocular Manifestations at Diagnosis of Anti-Neutrophil Cytoplasmic Anti-body-Associated Vasculitis: Clinical İmplications from a Retrospective Cohort

**DOI:** 10.1159/mpp/adpag011

**Published:** 2026-08-27

**Authors:** Merve Sungur Özgünen, Emre Tekgöz, Seda Çolak, Özlem Kılıç, Yusuf Uysal, Muhammet Çınar, Sedat Yılmaz

**Affiliations:** a Department of Rheumatology, University of Health Sciences, Gülhane Training and Research Hospital, Ankara, Turkey; b Department of Ophthalmology, University of Health Sciences, Gülhane Training and Research Hospital, Ankara, Turkey

**Keywords:** Anti-neutrophil cytoplasmic antibody-associated vasculitis, Granulomatosis with polyangiitis, Ocular involvement, Episcleritis, Scleritis, Multidisciplinary approach

## Abstract

**Objective:**

Ocular involvement is an important source of morbidity in anti-neutrophil cytoplasmic antibody (ANCA)-associated vasculitis (AAV) and may occasionally be the initial manifestation. This study evaluated the frequency, clinical characteristics, and systemic associations of ocular involvement in patients with AAV.

**Methods:**

In this retrospective cohort study, 105 patients with AAV followed at the Division of Rheumatology, University of Health Sciences Gülhane Training and Research Hospital between November 2016 and July 2024 were analyzed. Demographic, clinical, ANCA profile, vasculitis subtype, and organ involvement data were obtained from medical records. Patients were grouped according to ocular involvement at diagnosis, and clinical and laboratory characteristics were compared.

**Results:**

Ocular involvement was identified in 22 patients (21.0%). Twenty-one patients (95.5%) had granulomatosis with polyangiitis, and 18 (81.8%) were proteinase 3 anti-neutrophil cytoplasmic antibody-positive. Episcleritis (*n* = 12, 54.5%) and scleritis (*n* = 4, 18.2%) were the most common manifestations. Less frequent findings included anterior uveitis (*n* = 4, 18.2%), ischemic optic neuropathy (*n* = 1, 4.5%), and peripheral ulcerative keratitis associated with scleritis (*n* = 1, 4.5%). No significant differences were observed in demographic characteristics, disease activity, ANCA subtype distribution, or systemic organ involvement between groups.

**Conclusion:**

Ocular involvement is clinically relevant in AAV at diagnosis and may represent the initial presentation of systemic vasculitis. Episcleritis and scleritis were the most common findings, whereas vision-threatening complications were uncommon. Early recognition and ophthalmology-rheumatology collaboration may facilitate timely diagnosis and management.

**Highlights of the Study**

- Ocular involvement was identified in 21% of patients with anti-neutrophil cytoplasmic antibody-associated vasculitis at diagnosis.
- Granulomatosis with polyangiitis predominated among patients with ocular manifestations.
- Episcleritis and scleritis were the most common ocular findings, whereas vision-threatening complications were rare.
- Ocular symptoms may be the first manifestation of systemic vasculitis, highlighting ophthalmology-rheumatology collaboration.

## Introduction

Anti-neutrophil cytoplasmic antibody (ANCA)-associated vasculitis (AAV) comprises a group of chronic, systemic autoimmune diseases characterized by necrotizing inflammation of small and medium-sized blood vessels and frequently associated with circulating ANCA positivity [1]. AAV includes granulomatosis with polyangiitis (GPA), microscopic polyangiitis (MPA), and eosinophilic granulomatosis with polyangiitis, which are clinically heterogeneous disorders capable of affecting multiple organ systems, particularly the lungs, kidneys, upper respiratory tract, skin, and nervous system [2, 3].

Ocular and orbital manifestations have long been recognized as important features of AAV and are reported most frequently in patients with GPA [4]. The spectrum of ocular involvement ranges from relatively mild conditions such as episcleritis to potentially vision-threatening manifestations including scleritis, retinal vasculitis, optic neuropathy, and peripheral ulcerative keratitis [4–7]. Because the eye is one of the few organs in which vascular inflammation may be directly observed, ophthalmological examination may provide valuable diagnostic information in patients with systemic vasculitis [5, 6]. Ocular involvement in AAV is clinically important not only because of its potential to cause visual morbidity, but also because ocular symptoms may occasionally represent the first manifestation of systemic disease [7]. Early recognition of ocular findings may therefore facilitate timely diagnosis and treatment. Furthermore, the management of inflammatory ocular disease frequently requires close collaboration between ophthalmologists and rheumatologists [8].

Although ocular involvement in AAV has been described in previous studies, data specifically addressing ocular manifestations at the time of diagnosis remain limited. Therefore, this study aimed to evaluate the frequency, clinical characteristics, autoantibody profiles, systemic organ involvement, and treatment patterns of patients with AAV presenting with ocular manifestations at diagnosis and to compare them with patients without ocular involvement. In addition, we sought to highlight the importance of a multidisciplinary diagnostic approach involving ophthalmology and rheumatology.

## Subjects and Methods

This retrospective cohort study included consecutive patients with GPA and MPA who were followed at the Division of Rheumatology, University of Health Sciences Gülhane Training and Research Hospital between November 2016 and July 2024. Patients were identified from the institutional vasculitis database. Diagnoses were originally established according to the European Medicines Agency (EMA) algorithm [9] and the 2012 Chapel Hill Consensus Conference (CHCC) nomenclature [3], together with clinical findings, ANCA serology, and histopathological findings when available. For the purposes of the present study, all cases were subsequently re-evaluated according to the 2022 American College of Rheumatology/European Alliance of Associations for Rheumatology (ACR/EULAR) classification criteria [10, 11] by experienced rheumatologists. All consecutive eligible patients were included regardless of the presence or absence of ocular manifestations. Ophthalmological evaluation was not an inclusion criterion. In routine clinical practice, ophthalmology consultation was requested only for patients presenting with ocular symptoms or signs suggestive of ocular involvement, and ocular involvement was confirmed based on ophthalmological examination findings documented in ophthalmology clinic records. Presenting ocular symptoms leading to referral, including ocular redness, eye pain, foreign body sensation, blurred vision, photophobia, and visual impairment, were recorded when available. Ophthalmic manifestations included episcleritis, scleritis, anterior uveitis, retinal vasculitis, optic neuropathy, orbital involvement, peripheral ulcerative keratitis, and other ocular complications. Given the potential bidirectional referral between ophthalmology and rheumatology in patients presenting with ocular or systemic manifestations, the multidisciplinary diagnostic pathway is schematically illustrated in Figure 1. Therefore, the frequency of ocular involvement was calculated using the entire AAV cohort rather than a selected ophthalmology-referred subgroup.

**Fig. 1.**
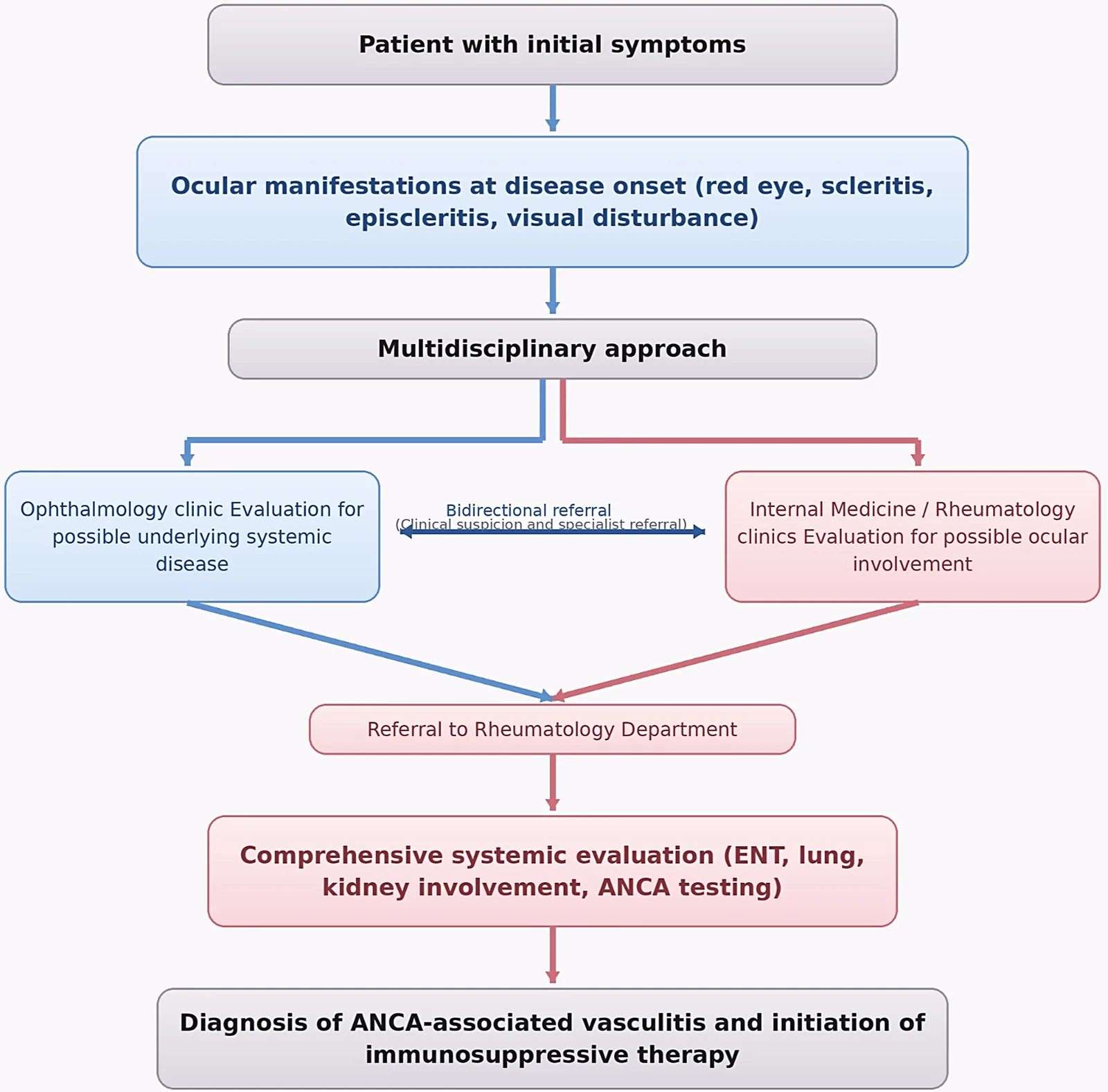
Multidisciplinary diagnostic pathway in patients with ocular manifestations of ANCA-associated vasculitis. Schematic illustration of the multidisciplinary diagnostic pathway in patients with ANCA-associated vasculitis presenting with ocular manifestations. Patients may initially present either to ophthalmology clinics with ocular symptoms or to internal medicine/rheumatology clinics with systemic manifestations. Bidirectional referral between ophthalmology and internal medicine/rheumatology allows comprehensive evaluation of ocular and systemic involvement, facilitating timely diagnosis and initiation of appropriate immunosuppressive therapy.

Patients with insufficient clinical data preventing confirmation of diagnosis, ANCA subtype, organ involvement status, or ocular involvement status were excluded. Demographic characteristics, ANCA subtypes (proteinase 3 [PR3]-ANCA and myeloperoxidase), clinical and laboratory findings, organ involvement, Birmingham Vasculitis Activity Score (BVAS), Five-Factor Score (FFS), induction and maintenance treatment regimens, ophthalmological findings, and follow-up data were extracted from electronic medical records. Systemic disease activity at diagnosis was assessed using the BVAS, and prognosis was evaluated using the FFS.

### Study Design and Comparisons

In the first stage of the analysis, patients with ocular involvement were compared with those without ocular involvement in terms of age, sex, ANCA subtype, vasculitis subtype, organ involvement, BVAS, FFS, and mortality rates. In the second stage, patients with documented ocular involvement were evaluated separately, with detailed assessment of demographic characteristics, underlying vasculitis diagnosis, extra-ocular manifestations, ANCA subtype, treatment regimens, and follow-up duration.

### Statistical Analysis

All statistical analyses were performed using IBM SPSS Statistics software. The distribution characteristics of continuous variables were assessed using the Kolmogorov–Smirnov and Shapiro–Wilk tests. Descriptive statistics were presented as mean ± standard deviation for normally distributed continuous variables and as median (interquartile range [IQR]) for non-normally distributed continuous variables. Categorical variables were presented as frequency and percentage. For intergroup comparisons, continuous variables were compared using the independent samples *t* test or Mann–Whitney U test, as appropriate, while categorical variables were compared using the Chi-square test or Fisher’s exact test, when appropriate. The level of statistical significance was set at *p* < 0.05.

## Results

Of the 105 patients with AAV included in the study, 57 (54.3%) were male and 48 (45.7%) were female. The mean age at diagnosis was 48.2 ± 15.3 years, and the median follow-up duration was 44 months (21.5–72.0 months). Ocular involvement was identified in 22 patients (21.0%). Of these patients, 13 (59.1%) were male and 9 (40.9%) were female, with a mean age at diagnosis of 45.9 ± 12.4 years. The demographic characteristics, AAV subtypes, ANCA profiles, disease activity measures, relapse rates, and mortality data of patients with and without ocular involvement are summarized in Table 1. Among patients with ocular involvement, 21 (95.5%) were diagnosed with GPA and 1 (4.5%) with MPA, whereas among patients without ocular involvement, 67 (80.7%) had GPA and 16 (19.3%) had MPA. No significant difference was observed in the distribution of AAV subtypes between the groups (*p = 0.082*). Similarly, ANCA subtype distribution did not differ significantly between patients with and without ocular involvement (*p = 0.442*). No significant differences were observed in the frequencies of pulmonary, upper respiratory tract, mucocutaneous, renal, joint, or neurological involvement between patients with and without ocular manifestations (all *p* > 0.05) (Table 1). The median BVAS was 7.0 (IQR 4.3) in patients with ocular involvement and 6.0 (IQR 5.0) in those without ocular involvement. The median FFS was 1.0 (IQR 1.3) and 1.0 (IQR 2.0), respectively. No significant differences were observed in BVAS (*p = 0.731*) or FFS (*p* = 0.515) between the groups. Likewise, relapse rates and mortality did not differ significantly between patients with and without ocular involvement (Table 1).

**Table 1.** Demographic and clinical characteristics of all AAV patients

Characteristic	All patients (*n* = 105)	Without eye involvement (%), (*n* = 83)	Eye involvement (%), (*n* = 22)	p value
Gender^a^ (Male)	57 (54.3)	44 (53.0)	13 (59.1)	0.611
Age at diagnosis^b^(mean±SD)	48.2±15.3	48.8±16.1	45.9±12.4	0.432
ANCA type^a^	Negative: 5 (4.8)	Negative: 4 (4.8)	Negative: 1 (4.5)	0.442
	PR3-ANCA: 75 (71.4)	PR3-ANCA: 57 (68.7)	PR3-ANCA: 18 (81.8)	
	MPO-ANCA: 25 (23.8)	MPO-ANCA: 22 (26.5)	MPO-ANCA: 3 (13.6)	
Diagnosis^c^	GPA: 88 (83.8)	GPA: 67 (80.7)	GPA: 21 (95.5)	0.082
	MPA: 17 (16.2)	MPA: 16 (19.3)	MPA: 1 (4.5)	
Pulmonary involvement^c^	83 (79.0)	64 (77.1)	19 (86.4)	0.264
Mucocutaneous involvement^c^	18 (17.1)	16 (19.3)	2 (9.1)	0.214
Respiratory tract involvement^c^	40 (38.1)	29 (34.9)	11 (50.0)	0.148
Renal involvement^c^	72 (68.6)	59 (71.1)	13 (59.1)	0.205
Joint involvement^a^	28 (26.7)	25 (30.1)	3 (13.6)	0.096
Neurological involvement^c^	12 (11.4)	9 (10.8)	3 (13.6)	0.481
BVAS^d^ (median – IQR)	6.0 (4.0–8.5)	6.0 (4.0–9.0)	7.0 (3.8–8.0)	0.731
FFS^d^ (median – IQR)	1.0 (0–2.0)	1.0 (0–2.0)	1.0 (0–1.3)	0.515
Relapse^a^	44.0 (41.9)	34.0 (41.0)	10.0 (45.5)	0.704
Mortality^c^	17.0 (16.2)	16.0 (19.3)	1.0 (4.5)	0.082

Continuous variables are presented as mean ± standard deviation or median (interquartile range), as appropriate. Categorical variables were compared using the chi-square test or Fisher’s exact test, depending on expected cell counts. Continuous variables were compared using the independent samples *t* test or Mann–Whitney U test, as appropriate.

^a^ Chi-square.

^b^ Independent samples *t* test.

^c^ Fisher’s exact test.

^d^ Mann-Whitney U.

The demographic characteristics, presenting ocular symptoms, ophthalmological diagnoses, extraocular manifestations, ANCA profiles, AAV subtypes, and treatment regimens of the 22 patients with ocular involvement are presented in Table 2. Episcleritis was the most common ocular manifestation, observed in 12 patients (54.5%), followed by scleritis in 4 (18.2%) and anterior uveitis in 4 (18.2%). Ischemic optic neuropathy and peripheral ulcerative keratitis associated with scleritis were each identified in 1 patient (4.5%) (Fig. 2). In 8 patients (36.4%), ocular symptoms constituted the initial presenting complaint and led to ophthalmology referral. Following ophthalmological evaluation, these patients were referred to rheumatology for further investigation, and subsequent systemic assessment established the diagnosis of AAV. Treatment regimens reflected the overall severity of systemic disease and organ involvement, while severe or vision-threatening ocular manifestations were also considered during treatment selection. The details of treatment regimens are also presented in Table 2.

**Table 2.** Demographic characteristics, ophthalmological diagnoses, extraocular involvement findings, ANCA profiles, AAV subtypes, and treatments of patients with ocular involvement at diagnosis

Patient number	Age, gender	Ocular complaint	First specialty consulted	Ophthalmological diagnosis	Extraocular involvement findings	ANCA profile	Diagnosis	Induction therapy	Maintenance therapy	Follow-up period
1	40, M	Ocular redness and foreign body sensation	Rheumatology	Episcleritis	SNHL, facial paralysis, respiratory tract involvement	Negative	GPA	Steroid + Cyc	MTX	168 months
2	36, M	Painful ocular redness and tearing	Ophthalmology	Scleritis	Pulmonary nodule	PR3 ANCA	GPA	Steroid + Cyc	MTX	264 months
3	36, M	Ocular redness and photophobia	Ophthalmology	Episcleritis	Pulmonary nodule, SNHL, skin rash, sinusitis, subglottic stenosis	PR3 ANCA	GPA	Steroid + Cyc	–	1 month
4	57, F	Ocular redness	Rheumatology	Episcleritis	Weight loss, sinusitis, pulmonary nodule, hematuria	PR3 ANCA	GPA	Steroid+ Cyc	AZA	72 months
5	42, M	Ocular redness	Rheumatology	Episcleritis	Skin rash, arthritis, nephritic proteinuria	PR3 ANCA	GPA	Steroid + Cyc	AZA	60 months
6	38, M	Ocular redness and foreign body sensation	Rheumatology	Episcleritis	Sinusitis, nephritic proteinuria, peripheral neuropathy	PR3 ANCA	GPA	Steroid + Cyc	AZA	72 months
7	41, M	Ocular redness and photophobia	Rheumatology	Episcleritis	Pulmonary nodule, sinusitis, nephritic proteinuria, cerebral and cerebellar ischemic gliotic foci	PR3 ANCA	GPA	Steroid + Cyc	AZA	64 months
8	25, F	Ocular redness, Photophobia and blurred vision	Rheumatology	Anterior uveitis	Arthritis, Nephritic proteinuria, and findings of lung parenchymal involvement	PR3 ANCA	GPA	Steroid + Cyc	AZA	180 months
9	65, M	Painful ocular redness and photophobia	Rheumatology	Scleritis	Findings of pulmonary parenchymal involvement, AKI, and Nephritic proteinuria	PR3 ANCA	GPA	Steroid + Cyc + Rituximab + Plasmapheresis	MMF	96 months
10	56, M	Ocular redness	Ophthalmology	Episcleritis	Pulmonary parenchymal involvement, sinusitis, AKI, nephritic proteinuria, and Arthritis	PR3 ANCA	GPA	Steroid + Cyc + Rituximab + Plasmapheresis	Leflunomide	40 months
11	35, F	Severe eye pain and coloured lump on the eye	Ophthalmology	Nodular scleritis	Sinusitis, pulmonary parenchymal involvement, nephritic proteinuria	PR3 ANCA	GPA	Steroid + Rituximab	Leflunomide	11 months
12	37, M	Ocular redness and foreign body sensation	Ophthalmology	Episcleritis	Pulmonary parenchymal involvement	PR3 ANCA	GPA	Steroid + Rituximab + MTX	MTX	7 months
13	65, F	Ocular redness	Rheumatology	Episcleritis	Pulmonary nodule, Sinusitis	PR3 ANCA	GPA	Steroid + MMF	MMF	17 months
14	24, M	Sudden vision loss	Ophthalmology	Right ischemic optic neuropathy	Pulmonary nodule, sinusitis	PR3 ANCA	GPA	Steroid + Rituximab + MTX	Rituximab + MTX	36 months
15	35, F	Ocular redness and photophobia	Ophthalmology	Anterior uveitis	Sinusitis	PR3 ANCA	GPA	Steroid + AZA	AZA	24 months
16	58, M	Severe nocturnal eye pain, photophobia, blurred vision	Ophthalmology	Peripheral ulcerative keratitis and scleritis	Pulmonary nodule, sinusitis	PR3 ANCA	GPA	Steroid + AZA	AZA	16 months
17	45, M	Painful ocular redness	Rheumatology	Scleritis	Pulmonary nodule, nephritic proteinuria, orchitis	PR3 ANCA	GPA	Steroid + Cyc + Rituximab	MMF	72 months
18	56, M	Ocular redness	Rheumatology	Episcleritis	Pulmonary nodule	PR3 ANCA	GPA	Steroid + MTX	MTX	4 months
19	47, F	Ocular redness, blurred vision	Rheumatology	Anterior uveitis	Pulmonary nodule, nephritic proteinuria	PR3 ANCA	GPA	Steroid + Cyc	MTX >> AZA	264 months
20	61, F	Ocular redness and foreign body sensation	Rheumatology	Episcleritis	Lung parenchymal involvement findings, AKI	MPO ANCA	MPA	Steroid + Cyc + Plasmapheresis	AZA	60 months
21	55, F	Ocular redness and foreign body sensation	Rheumatology	Episcleritis	Findings of lung parenchymal involvement	MPO ANCA	GPA	Steroid + Cyc	AZA	72 months
22	56, F	Ocular redness, photophobia	Rheumatology	Anterior uveitis	Pulmonary nodule, AKI	MPO ANCA	GPA	Steroid + Cyc + Rituximab + Plasmapheresis	MMF	84 months

F, female; M, male; SNHL, sensorineural hearing loss; GPA, granulomatous polyangiitis; MPA, microscopic polyangiitis; Cyc, cyclophosphamide; AKI, acute kidney injury; AZA, azatyoprine; MTX, methotrexate; MMF, mycophenolate mofetil.

**Fig. 2.**
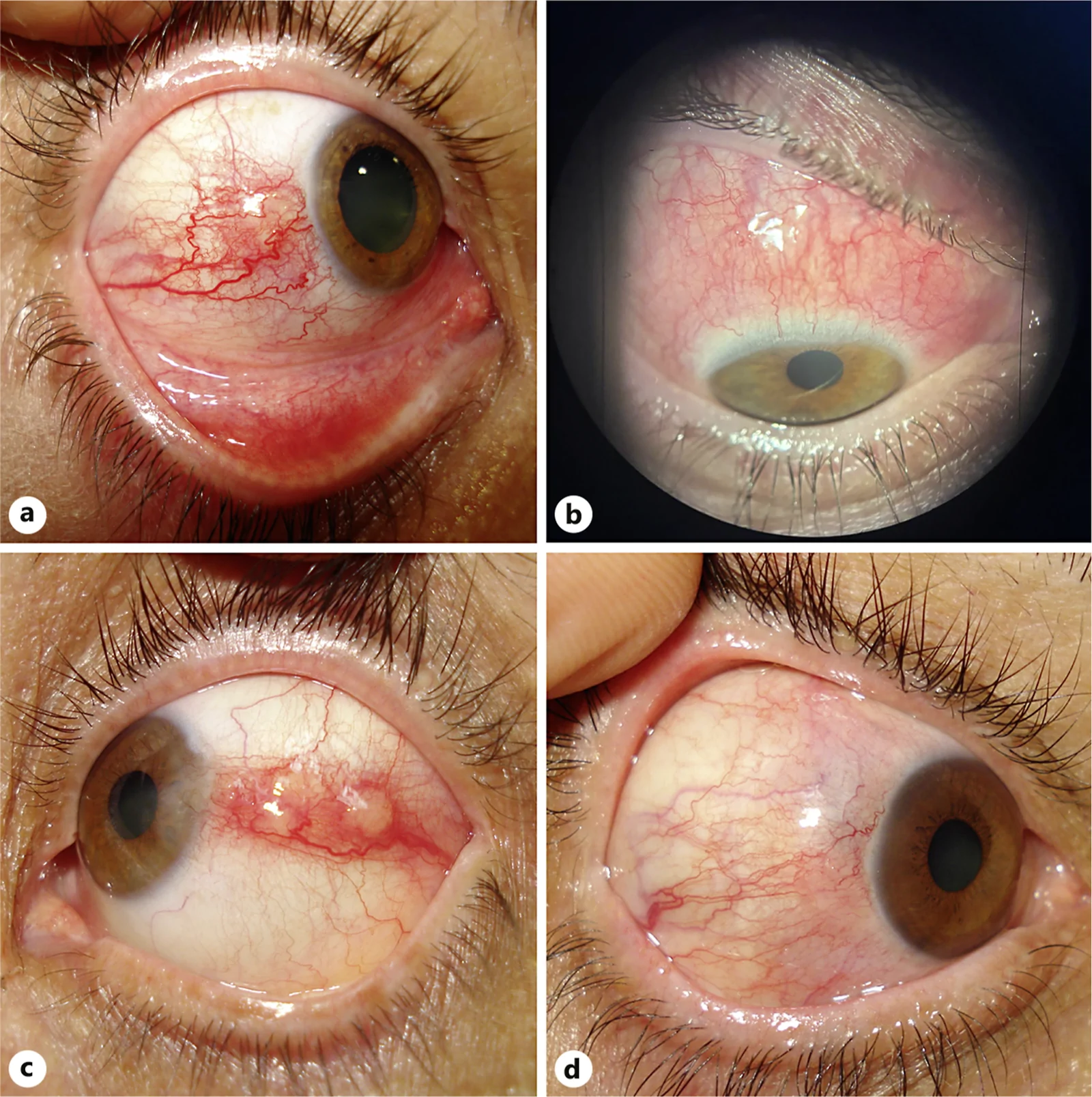
Ocular manifestations in ANCA-associated vasculitis patients. **a** Nodular episcleritis. **b** Diffuse scleritis. **c** Nodular scleritis and peripheral ulcerative keratitis. **d** Scleritis.

## Discussion

This study provides clinically meaningful data from a cohort of 105 patients with AAV, including detailed characterization of ocular manifestations at diagnosis and treatment patterns in a tertiary referral center. From a rheumatology perspective, while pulmonary and renal involvement are often emphasized in daily practice, ocular involvement represents an important source of morbidity and may occasionally constitute the initial manifestation of the disease. Moreover, ocular conditions with a potential risk of vision loss may directly influence treatment decisions, highlighting their clinical relevance [1, 2, 4]. In our cohort, ocular involvement was identified in 21% of patients at the time of diagnosis, a finding consistent with previously reported rates in the literature [12–14]. Although long-term follow-up of ocular manifestations was not available in our study, previous reports suggest that the frequency of ocular involvement may increase during the chronic course of AAV [12–14]. These findings emphasize the importance of maintaining awareness of ocular symptoms throughout the disease course.

GPA accounted for the vast majority of patients with ocular involvement in our study, whereas MPA was observed in only 1 patient. Although neither GPA frequency nor PR3-ANCA positivity differed significantly between patients with and without ocular involvement, previous studies have consistently reported a predominance of ocular manifestations among patients with GPA and PR3-ANCA positivity [15–17]. A similar distribution was observed in our cohort, although these differences did not reach statistical significance.

No significant differences were observed in systemic organ involvement between patients with and without ocular manifestations. Previous studies have reported associations between ocular disease and sinonasal, orbital, or central nervous system involvement [12, 18, 19]. However, such relationships were not observed in our cohort, suggesting that ocular manifestations may occur across a broad spectrum of disease presentations and are not necessarily linked to specific patterns of systemic organ involvement.

Consistent with previous reports, episcleritis and scleritis were the most common ocular diagnoses observed in our cohort [12, 14, 20, 21]. Although less frequent, anterior uveitis, ischemic optic neuropathy, and peripheral ulcerative keratitis were also identified. Vision-threatening manifestations were uncommon, occurring in only 2 patients; however, these conditions remain clinically important because they may be associated with significant morbidity and irreversible visual loss [18, 22, 23]. Accordingly, prompt recognition and treatment of ocular involvement remain essential components of AAV management.

Treatment strategies in our cohort were primarily determined by the extent and severity of systemic disease and major organ involvement rather than by ocular manifestations alone. Accordingly, many patients with episcleritis received cyclophosphamide- or rituximab-based induction therapy because of concomitant pulmonary, renal, or other organ-threatening disease. However, severe vision-threatening ocular manifestations could also influence treatment selection. For example, the patient with ischemic optic neuropathy received rituximab-based induction therapy because of the high risk of irreversible visual loss. These findings highlight that although treatment in AAV is largely guided by overall disease severity, the presence of sight-threatening ocular involvement may justify escalation of systemic immunosuppressive therapy. Consequently, close collaboration between rheumatologists and ophthalmologists remains essential for optimal treatment planning [24–26].

Notably, 8 patients (36.4%) with ocular involvement in our cohort initially presented to ophthalmology clinics and were subsequently referred to rheumatology, where the diagnosis of AAV was established following the identification of additional systemic findings. This observation highlights the pivotal role of ophthalmologists in the early recognition of systemic vasculitis and underscores the importance of interdisciplinary collaboration in the diagnostic process [27, 28]. The identification of ocular symptoms as the initial manifestation of AAV further emphasizes that systemic vasculitis should be considered in the differential diagnosis of inflammatory ocular disease.

### Limitations and Strengths

Several limitations of this study should be acknowledged. First, the retrospective design may have resulted in incomplete data capture. Second, the lack of detailed ophthalmological examination findings and longitudinal ophthalmological follow-up limited our ability to comprehensively characterize the evolution and outcomes of ocular manifestations. Third, as this study was conducted at a tertiary referral center, referral bias toward patients with more severe disease manifestations cannot be excluded. Finally, the single-center design may limit the generalizability of our findings.

Despite these limitations, the relatively large sample size represents a notable strength for a single-center cohort of AAV patients. In addition, the identification of patients in whom ocular manifestations constituted the initial presentation of vasculitis, prompting referral from ophthalmology to rheumatology and leading to the diagnosis of AAV, represents an important strength of this study. This finding reinforces the clinical value of close collaboration between ophthalmologists and rheumatologists in the timely diagnosis and management of systemic vasculitis.

## Conclusion

Ocular involvement is a common manifestation of ANCA-associated vasculitis and may represent the initial clinical presentation in a subset of patients, contributing substantially to disease-related morbidity. In our cohort, ocular involvement occurred predominantly in patients with GPA and PR3-ANCA positivity. Episcleritis and scleritis were the most common ocular manifestations, whereas vision-threatening complications were uncommon but clinically important. Early recognition of ocular findings and a multidisciplinary approach involving rheumatology and ophthalmology may facilitate timely diagnosis and appropriate management of AAV. Further prospective multicenter studies are warranted to better define the clinical implications of ocular involvement throughout the course of ANCA-associated vasculitis.

## Statement of Ethics

All data were recorded in a standardized data collection form, and the study was conducted in accordance with the principles of the Declaration of Helsinki. Ethical approval for the study was obtained from the University of Health Sciences Gülhane Training and Research Hospital Non-Interventional Scientific Research Ethics Committee (Approval No.: 2024/39). Due to the retrospective design of the study, the requirement for written informed consent was waived by the ethics committee.

## Conflict of Interest Statement

The authors declare no conflicts of interest.

## Funding Sources

The authors declare no funding was obtained or used for this study.

## Author Contributions

Merve Sungur Özgünen and Emre Tekgöz contributed to the conceptualization and methodology of the study. Formal analysis and investigation were performed by Merve Sungur Özgünen and Özlem Kılıç. Merve Sungur Özgünen drafted the original version of the manuscript. Emre Tekgöz, Muhammet Çınar, and Sedat Yılmaz critically revised the manuscript for important intellectual content. Seda Çolak and Yusuf Uysal supervised the study. All authors read and approved the final manuscript.

## Data Availability

The data that support the findings of this study are available on request from the corresponding author. The data are not publicly available due to privacy or ethical restrictions.
